# Assessment of in vivo metabolism in failing hearts using hyperpolarised 13C magnetic resonance

**DOI:** 10.1186/1532-429X-13-S1-O79

**Published:** 2011-02-02

**Authors:** Marie A Schroeder, Angus Lau, Albert Chen, Kim Connelly, Jennifer Barry, Kieran Clarke, Graham Wright, Charles Cunningham

**Affiliations:** 1 Sunnybrook Research Institute, Toronto, ON, Canada; 2 Department of Medical Biophysics, University of Toronto, Toronto, ON, Canada; 3 eenan Research Centre of the Li Ka Shing Knowledge Institute, St. Michael's Hospital, Toronto, ON, Canada; 4 University of Oxford, Oxford, UK

## Introduction

Increasingly, abnormal metabolic substrate utilisation is considered a cause of heart failure (HF). Hyperpolarised ^13^C MR, a technique in which the fate of ^13^C-labelled metabolites can be followed *in vivo* using MR imaging or spectroscopy, has enabled non-invasive assessment of cardiac substrate utilisation.

## Purpose

The aim of this study was to monitor carbohydrate metabolism alongside cardiac structure and function, throughout HF progression.

## Methods

Dilated cardiomyopathy (DCM) was induced in pigs (n = 4) by rapid ventricular pacing at 188 bpm for 4-5 weeks. Pigs were examined at baseline and at weekly time points throughout DCM progression. At each time point, cine MRI was used to assess cardiac structure and function, 0.05 mmol/kg hyperpolarised ^13^C_2_-pyruvate was administered intravenously and MRS was used to assess Krebs cycle-mediated ^13^C-glutamate production, and hyperpolarised ^13^C_1_-pyruvate was administered to assess H^13^CO_3_^-^ production from pyruvate dehydrogenase (PDH), and thus relative carbohydrate oxidation. A new cardiac and respiratory-gated ^13^C MRI sequence was used to image ^13^C_1_-pyruvate and H^13^CO_3_^-^. The chemical shift-specific pulse sequence used allowed temporally resolved imaging of ^13^C_1_-pyruvate and H^13^CO_3_^-^ with 9 mm in-plane spatial resolution in multiple slices (two in these studies), all within a 23 s scan. Pigs were sacrificed after presentation of clinical symptoms or >25% increase in end diastolic volume (EDV).

## Results

**Figure 1:**
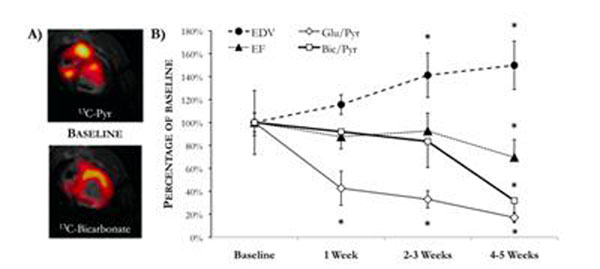
A) Representative short-axis ^13^C images of the healthy pig heart, acquired with a surface coil. B) Comparison of parameters of structural and metabolic remodeling in the pig heart throughout the progression of DCM. *p<0.05.

At baseline, pigs had an EDV of 62 ± 5 ml. The maximum ^13^C-glutamate/^13^C_2_-pyruvate ratio was 4.9 ± 1.2% (Fig 1A), whereas the mean H^13^CO_3_^-^/^13^C_1_-pyruvate ratio across the anterior myocardium was 2.0 ± 0.3% (Fig 1B). After 1 week of pacing, the ^13^C-glutamate/^13^C_2_-pyruvate decreased significantly to 2.1 ± 0.8%, and was maintained at this level throughout DCM development. EDV increased linearly with pacing duration, and after 2-3 weeks of pacing was significantly elevated to 84 ± 12 ml. After 4-5 weeks of pacing (at the final time point), the ejection fraction (EF) was decreased by 40% compared with the baseline value, and the H^13^CO_3_^-^/^13^C_1_-pyruvate was decreased to 0.8±0.2%.

## Conclusions

In conclusion, metabolism of ^13^C_2_-pyruvate to ^13^C-glutamate was reduced by 59% at an early stage in DCM, with no change to PDH flux. Reduced ^13^C-glutamate relative to H^13^CO_3_^-^ production could be an early marker of disease. Carbohydrate oxidation via PDH was maintained until end-stage DCM, at which point PDH flux was reduced by 62%. With further development, metabolic imaging using hyperpolarised ^13^C MR may similarly characterize HF progression in patients.

